# Significant Stenosis of the Brachiocephalic Trunk and Moderate Stenosis of the Left Circumflex Artery in Computed Tomography Angiography Images

**DOI:** 10.3390/diagnostics12010200

**Published:** 2022-01-14

**Authors:** Paweł Gać, Rafał Poręba

**Affiliations:** 1 Centre for Diagnostic Imaging, 4th Military Hospital, Weigla 5, PL 50-981 Wroclaw, Poland; 2 Department of Population Health, Division of Environmental Health and Occupational Medicine, Wroclaw Medical University, Mikulicza-Radeckiego 7, PL 50-368 Wroclaw, Poland; 3 Department of Internal and Occupational Diseases, Hypertension and Clinical Oncology, Wroclaw Medical University, Borowska 213, PL 50-556 Wroclaw, Poland; rafal.poreba@umw.edu.pl

**Keywords:** brachiocephalic trunk stenosis, computed tomography angiography, moderate coronary artery stenosis, steal syndrome

## Abstract

Atherosclerosis, as a civilization disease, is a serious epidemiological problem. Significant carotid disease and significant coronary artery disease result in acute consequences, such as ischemic stroke and myocardial infarction, which are the major causes of cardiovascular mortality. Typically, atherosclerosis of the aortic arch branches involves the bulbs of the common carotid arteries and the proximal segments of the internal carotid arteries, and can be effectively assessed by ultrasonography. Computed tomography angiography enables the identification of patients with less typical clinical manifestations of atherosclerosis, e.g., brachiocephalic trunk stenosis with symptoms of the steal syndrome and moderate stenosis in the coronary arteries. We present examples of computed tomography angiography images of this type of changes.

A 74-year-old male was referred to a cardiology outpatient clinic for further evaluation of a transient loss of consciousness.

The medical history revealed arterial hypertension, type two diabetes, previous rheumatic disease (14–15 years), and revascularization of the right lower limb arteries. The patient reported an episode of syncope during housework. The syncope episode occurred when the patient raised his arms upward during the work. The patient suffered from balance disorders, dizziness, and numbness in the upper limbs.

Physical examination results: good circulatory and respiratory function, constant HR at 70 bpm, right brachial BP 125/65 mmHg, left brachial BP 130/70 mmHg, and small varicose veins in the left shin.

Ultrasound examination of the carotid arteries revealed bilateral atherosclerotic plaques in the visualized carotid arteries sections. A Doppler examination revealed significantly lower flow velocities in the carotid arteries on the right side compared to the contralateral side.

Due to the reported non-specific cardiovascular complaints and the ultrasound image of the carotid arteries, the patient was referred to the computed tomography (CT) laboratory for computed tomography angiography (CTA) of the coronary arteries and computed tomography of the carotid and vertebral arteries.

The CTA of the coronary arteries, performed using a 384-slice Siemens Force CT, revealed a coronary artery calcium score of 412.5 (left main (LM): 185, left anterior descending (LAD): 16.7, and left circumflex (LCx): 210.8), the value of which indicated a high risk of a significant coronary artery disease (Figure 1A); the examination revealed numerous atherosclerotic plaques of various morphotic types in the coronary arteries, causing short-segment stenosis, up to 50–70% within the left circumflex, right behind the origin of the 1. marginal branch (Figure 1B) and the long muscular bridge within the middle section of the left anterior descending, about 5.8 cm long (Figure 1C). The CTA images of the coronary arteries corresponded to level three in the CAD-RADS classification, meaning a moderate coronary disease with the presence of moderate LCx stenosis requiring an objective evaluation, optimally via functional tests [1].

The CTA of the carotid and vertebral arteries performed using a 384-slice Siemens Force CT revealed a typical origin system of the aortic arch branch, numerous atherosclerotic plaques of various morphotic types in the aortic arch and its branches, significant ostial stenosis of the brachiocephalic trunk by 70–90% of the vessel lumen (Figure 1D), recessive right vertebral artery (Figure 1E), and ostial insignificant stenosis of the left subclavian artery (Figure 1F). In the CTA the right carotid arteries and the right cerebral arteries were characterized by a significantly lower lumen density compared to the contralateral side (Figure 1G,H), which may indicate impaired flow or its direction reversal. The CTA image of the carotid and vertebral arteries corresponded to significant stenosis of the brachiocephalic trunk with symptoms of the steal syndrome.

The patient was qualified for further diagnostics and treatment. Further cardiological diagnostics were planned to objectively evaluate the stenosis in the left circumflex, in addition to a consultation with a vascular surgeon to consider invasive treatment of the significant stenosis in the brachiocephalic trunk.

The complaints reported by the patient could be most likely related to the steal syndrome, which intensified when the patient raised his right arm. Such an explanation of the syncope episode is indicated by the summary of all data from the anamnesis (syncope episode when the patient raised his arms, i.e., when there was a compensatory increase in the reversal of the flow direction in the carotid arteries and subsequent cerebral hypoperfusion), physical examination (lower values of the resting blood pressure measured on the right arm compared to the measurement on the left arm), and imaging examinations (significantly lower flow velocities in the right carotid arteries in the carotid ultrasound and significantly lower lumen density of the right carotid arteries and the right cerebral arteries in the carotid CTA). Blood pressure values were only slightly lower on the right arm than on the left arm. However, it should be remembered that, physiologically, blood pressure values measured on the right arm should be higher; the measurements taken were resting measurements made in the standard position of the upper limbs. Moreover, the patient was treated with antihypertensive drugs due to the diagnosed arterial hypertension.

The steal syndrome resulting from flow disorders in the brachiocephalic trunk is a relatively rare type of this type of pathology. Most often, the steal syndrome affects the left subclavian artery and results from the narrowing of its proximal part [2]. In the articles published so far there are few case reports of the brachiocephalic steal syndrome [3,4,5,6]. In summary, they note that, in contrast to the subclavian steal, brachiocephalic trunk stenosis induces distinct and much more significant hemodynamic alterations in extracranial arterial flow and is rarely asymptomatic. Symptoms of significant right-sided cerebrovascular insufficiency as well as progressive pain and weakness of the right upper extremity were the most common causes of diagnosis when brachiocephalic steal syndrome was diagnosed [3,4,5,6].

The relationship between the degree of coronary stenosis and myocardial ischemia is one of the key diagnostic problems of coronary artery disease [7]. Currently, it is assumed that stenosis < 50% does not result in myocardial ischemia, and thus does not require further diagnostic and intervention procedures. Stenoses > 70% usually cause such myocardial ischemia, which translates into the need for invasive coronary diagnosis with FFR assessment and invasive coronary treatment when ischemia is confirmed in FFR [8,9]. Stenoses of 50–70% are problematic as they may result in myocardial ischemia in some patients, while in other patients they do not have to cause such ischemia. As indicated in the CAD-RADS classification, 50–70% stenoses require additional functional assessment, i.e., stress tests with adenosine/regadenoson [10,11].

## Figures and Tables

**Figure 1:**
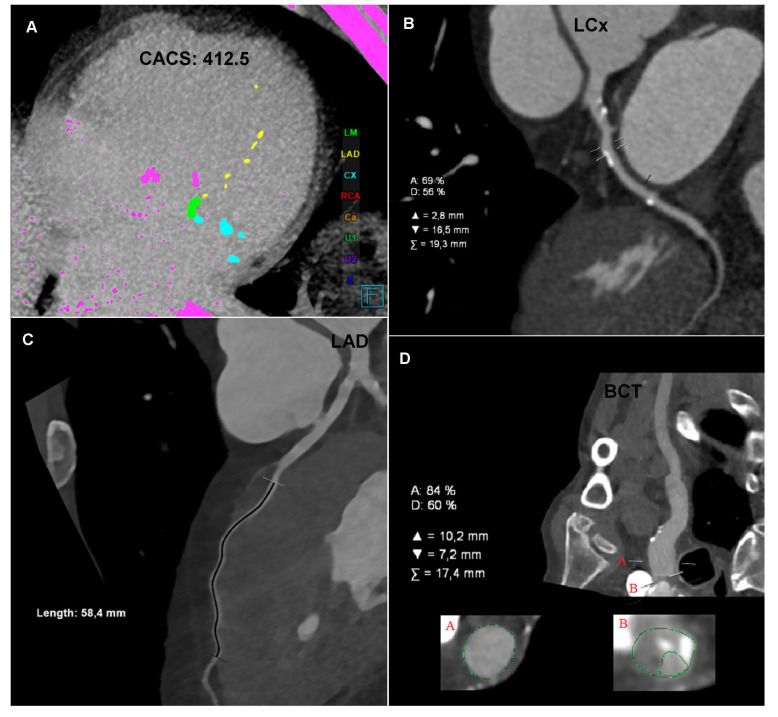

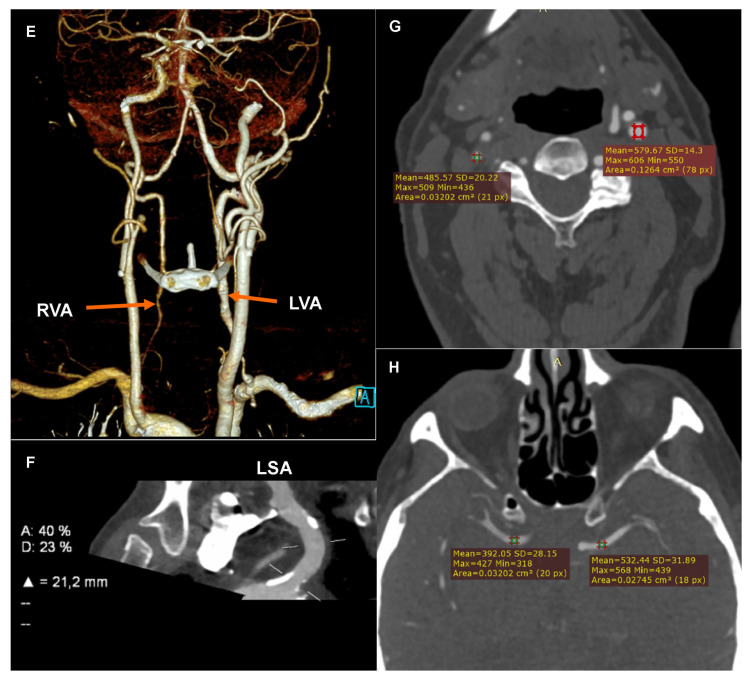
Computed tomography angiography (CTA): (**A**) Coronary CTA. Native phase. Axial reconstruction. Coronary artery calcium score measurement. The colors indicate the calcifications assigned to specific coronary arteries. (**B**) Coronary CTA. Angiographic phase. Curved multiplanar reconstruction (cMPR). Left circumflex (LCx). Measurement of the degree of stenosis. (**C**) Coronary CTA. Angiographic phase. Curved multiplanar reconstruction (cMPR). Left anterior descending (LAD). Measurement of the length of the myocardial muscle bridge. (**D**) Carotid CTA. Angiographic phase. Curved multiplanar reconstruction (cMPR). Brachiocephalic trunk (BCT). Measurement of the degree of ostial stenosis. The letter A indicates the level of vessel lumen measurement at the reference level, the letter B indicates the level of vessel lumen measurement at the level of maximum stenosis. Computed tomography angiography (CTA): (**E**) Carotid CTA. Angiographic phase. Volume rendering technique reconstruction (VRT). Arrows mark the recessive right vertebral artery (RVA) and the dominant left vertebral artery (LVA). (**F**) Carotid CTA. Angiographic phase. Curved multiplanar reconstruction (cMPR). Left subclavian artery (LSA). Measurement of the degree of stenosis. (**G**) Carotid CTA. Angiographic phase. Axial reconstruction. Measurement of the density of the proximal sections of the internal carotid arteries. Lower density of contrasted blood in the right carotid arteries. (**H**) Carotid CTA. Angiographic phase. Axial reconstruction. Measurement of the density of the cerebral arteries. Lower density of contrasted blood in the right cerebral arteries.
